# Myocardial Inflammation as a Manifestation of Genetic Cardiomyopathies: From Bedside to the Bench

**DOI:** 10.3390/biom13040646

**Published:** 2023-04-04

**Authors:** Giovanni Peretto, Elena Sommariva, Chiara Di Resta, Martina Rabino, Andrea Villatore, Davide Lazzeroni, Simone Sala, Giulio Pompilio, Leslie T. Cooper

**Affiliations:** 1 Department of Cardiac Electrophysiology and Arrhythmology, IRCCS San Raffaele Scientific Institute, 20132 Milan, Italy; 2 School of Medicine, Vita-Salute San Raffaele University, 20132 Milan, Italy; 3 Unit of Vascular Biology and Regenerative Medicine, Centro Cardiologico Monzino IRCCS, 20139 Milan, Italy; 4 Genomic Unit for the Diagnosis of Human Pathologies, IRCCS San Raffaele Scientific Institute, 20132 Milan, Italy; 5 IRCCS Fondazione Don Carlo Gnocchi, 43100 Parma, Italy; 6 Department of Biomedical, Surgical and Dental Sciences, Università degli Studi di Milano, 20122 Milan, Italy; 7 Department of Cardiovascular Medicine, Mayo Clinic, Jacksonville, FL 32224, USA

**Keywords:** myocardial inflammation, cardiomyopathies, genetics, preclinical models, bench, dilated, arrhythmogenic, ventricular arrhythmias, desmosomes, sudden cardiac death, endomyocardial biopsy, cardiac magnetic resonance

## Abstract

Over recent years, preclinical and clinical evidence has implicated myocardial inflammation (M-Infl) in the pathophysiology and phenotypes of traditionally genetic cardiomyopathies. M-Infl resembling myocarditis on imaging and histology occurs frequently as a clinical manifestation of classically genetic cardiac diseases, including dilated and arrhythmogenic cardiomyopathy. The emerging role of M-Infl in disease pathophysiology is leading to the identification of druggable targets for molecular treatment of the inflammatory process and a new paradigm in the field of cardiomyopathies. Cardiomyopathies constitute a leading cause of heart failure and arrhythmic sudden death in the young population. The aim of this review is to present, from bedside to bench, the current state of the art about the genetic basis of M-Infl in nonischemic cardiomyopathies of the dilated and arrhythmogenic spectrum in order to prompt future research towards the identification of novel mechanisms and treatment targets, with the ultimate goal of lowering disease morbidity and mortality.

## 1. Myocarditis and Primary Cardiomyopathies

### 1.1. Introduction

The World Health Organization defines myocarditis as an inflammatory disease of the myocardium diagnosed by established histological, immunological, and immunohistochemical criteria [1]. The main recognized etiologies of myocarditis include viral infections, toxic agents, and autoimmune mechanisms [2]. Although a genetic background has been hypothesized either as a predisposing [3] or accelerating factor [4], myocarditis is currently classified as a nongenetic disease [2]. Over the last decade, however, a growing body of evidence has attempted to identify a strict connection between genetics and myocarditis. In particular, pathogenic variants in cardiomyopathic genes have been identified in probands with familial myocarditis. On the other hand, bursts of active myocardial inflammation (M-Infl) [5] overlapping with classic myocarditis have been reported in patients with known genetic cardiomyopathies. Since the development of a manifest phenotype of each genetic disease depends on the interaction between genetics and environment, myocardial inflammation may represent the “cellular environment” that plays a primary role in the evolution of cardiomyopathies. The aim of this narrative review is to describe the state of the art regarding the genetic basis of myocarditis. In particular, the connection points between myocarditis and cardiomyopathies of the dilated and arrhythmogenic spectrum will be presented in both the clinical and preclinical settings. A brief overview of the current definitions of myocarditis and cardiomyopathies is presented in Table 1.

### 1.2. Diagnosis of Myocarditis

**Figure 1:**
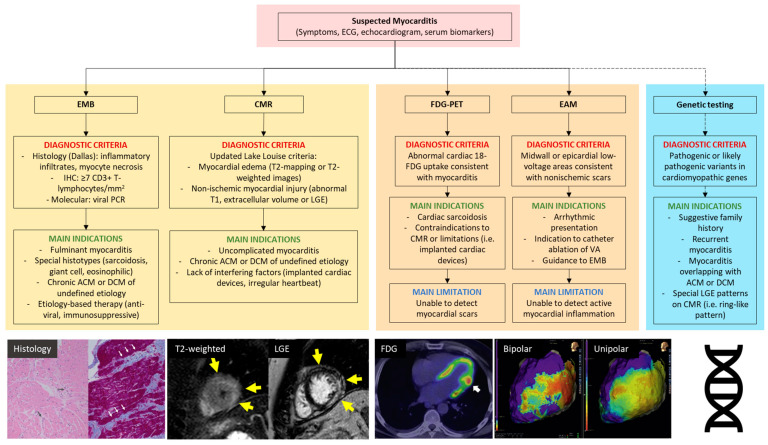
Diagnostic workup for myocarditis. The diagnostic workup for myocarditis is summarized, including gold standard techniques (EMB and CMR, yellow panel), additional imaging techniques applying to special patient subsets (FDG-PET and EAM, orange panel), and genetic testing to investigate the overlap with primary cardiomyopathies (blue panel). For each diagnostic exam, diagnostic criteria and main indications are reported. In each figure, the arrows point to remarkable findings. Representative examples are shown in the lower panel. ACM = arrhythmogenic cardiomyopathy; CMR = cardiac magnetic resonance; DCM = dilated cardiomyopathy; EAM = electroanatomic mapping; EMB = endomyocardial biopsy; FDG-PET = ^18^F-fluorodeoxypositron emission tomography; IHC = immunohistochemistry; LGE = late gadolinium enhancement; PCR = polymerase chain reaction; VA = ventricular arrhythmias.

Based on the aforementioned definition and updated evidence [6,7], histology is the gold standard of diagnostics for myocarditis. In particular, the European Society of Cardiology (ESC) proposed specific histological and immunohistochemical criteria [6,8], reported in Table 1. In this setting, borderline myocarditis, defined as the absence of myocyte necrosis [6], is frequently indistinguishable from cardiomyopathy-associated M-Infl [5] (Table 2). Histology information can be obtained by means of endomyocardial biopsy (EMB), provided that tissue sampling is adequate and informative [6,15]. As a diagnostic tool complementary to EMB, cardiac magnetic resonance (CMR) allows noninvasive, multiplanar, and panoramic investigation of myocarditis [2]. In particular, the key pathophysiological components of myocarditis, namely hyperemia, edema, and necrosis, are mirrored by distinct CMR sequences. As defined by the updated Lake Louise criteria [16], while T2-weighted sequences point to M-Infl, late gadolinium enhancement (LGE) is observed both during the acute and the post-inflammatory stages of myocarditis [4]. In the chronic setting, LGE on CMR, as well as low-voltage areas on electro-anatomical mapping, are considered as rough equivalents of myocardial scar [5,9]. Among the other imaging techniques, ^18^F-fluorodeoxy-positron emission tomography (FDG-PET) constitutes an informative diagnostic test when CMR is not feasible, such as in cardiac device carriers [17]. The main techniques and diagnostic criteria for myocarditis are summarized in Figure 1.

### 1.3. Classification of Cardiomyopathies

**Figure 2:**
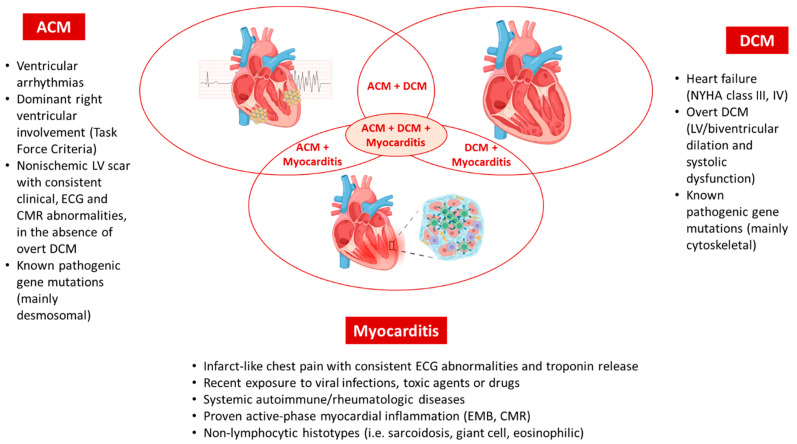
Overlap between myocarditis and primary cardiomyopathies. The Venn diagram shows the overlaps between myocarditis and genetic cardiomyopathies of the arrhythmogenic and dilated spectrums. ACM = arrhythmogenic cardiomyopathy; CMR = cardiac magnetic resonance; DCM = dilated cardiomyopathy; EMB = endomyocardial biopsy.

Even in the absence of universal agreement about their definition (Table 1), cardiomyopathies are defined as myocardial disorders in which the heart muscle is structurally and/or functionally abnormal in the absence of alternative explanatory causes, including coronary artery disease, hypertension, valvular, and congenital heart disease [10,11]. Among a wide spectrum of nonischemic muscle diseases, dilated cardiomyopathy (DCM) is characterized by dilation and impaired contraction of the left or both ventricles [12], and is caused by rare genetic variants in nearly half of cases [26]. On the contrary, arrhythmogenic cardiomyopathy (ACM), in turn caused by gene variants in up to 50% of familial cases [13], has arrhythmic manifestations as the main clinical phenotype. In clinical practice, DCM and ACM constitute the main source of overlap with myocarditis and inflammatory cardiomyopathy [6,12,27,28]. In detail, while heart failure manifestations call for differential diagnosis with DCM [6,12], ventricular arrhythmias (VA) may suggest either classic or left dominant ACM [13,14]. In both cases, the nonischemic pattern of LGE is frequently found on the CMR [9]. Figure 2 shows the whole spectrum of overlapping phenotypes of DCM, ACM, and myocarditis. Although, in the vast majority of cases, specific diagnostic criteria allow a conclusive differential diagnosis among DCM, ACM, and myocarditis, in some cases a definitive diagnosis represents an inconclusive challenge due to a substantial overlap between clinical, instrumental, laboratory, and anatomical-histological data (Figure 2).

## 2. Myocarditis as a Manifestation of Primary Cardiomyopathy: Clinical Scenarios

### 2.1. Familial Myocarditis

A family history of sudden death or cardiomyopathy is the main clue to suspect genetic cardiomyopathies [29]. However, a hereditary component has also been described for myocarditis [2]. In detail, it has been reported that genetic variants involved in familial cardiomyopathies are frequently found in subjects with childhood-onset disease [30] and EMB-proven lymphocytic myocarditis [31]. 

Among DCM-causing genes, lymphocytic myocarditis has been suggested as a driver for the disease progression [32] in patients with truncating variants of the titin gene (*TTN*), which constitute the most prevalent cause of familial DCM. Other cytoskeletal gene variants have been shown to increase susceptibility to viral myocarditis [33], accounting for inconstant viral clearance and heterogeneous evolution towards inflammatory DCM [31]. For instance, variants in genes encoding structural proteins such as *DMD* have been associated with the persistence of viral genomes and unfavorable outcomes [34,35], whereas variants affecting genes coding for non-structural proteins such as *SCN5A* and *BAG3* lead to slowly progressing disease [33]. In a recent series of genotyped probands, a higher prevalence of pathogenic or likely pathogenic variants, in particular in the *FLNC*, *RBM20,* and *BAG3* genes, was reported in association with severe forms of myocarditis, including cardiogenic shock and sustained VA [36].

As for ACM, pathogenic variants in desmosomal genes, namely plakoglobin (*JUP*), desmoplakin (*DSP*), plakophilin (*PKP2*), desmoglein (*DSG2*), and desmocollin (*DSC*), are depicted as the main cause [13], in turn displaying extensive overlap with myocarditis. A recent study reported variants in the *DSP* and *DSG2* genes in six probands with familial myocarditis [20], allowing subsequent identification of up to 28 gene variant carriers, 39% of whom had a phenotype consistent with LV rather than classic right ventricular (RV) ACM. Myocarditis has been recently included within the typical phenotype of desmoplakin cardiomyopathy [18], where VA and recurrence of acute myocarditis are frequently associated [21,22].

A further challenging topic for familial myocarditis is the overlap between genetic background and autoimmunity. Recently, either pathogenic or likely pathogenic variants in genes associated with recessive immune disorders have been identified in pediatric patients with myocarditis [37]. Consistently with the complex gene-environment interactions underlying the pathophysiology of inflammatory cardiomyopathy [2,6], serum anti-heart autoantibodies and anti-intercalated disk autoantibodies have been found in the majority of patients with familial ACM [38], as well as in arrhythmic myocarditis [39]. Additional evidence includes the finding of circulating anti-myosin and anti-troponin I autoantibodies in DSP cardiomyopathy [40] and of anti-DSG2 autoantibodies in classic RV-dominant ACM [41].

### 2.2. Autoptic Findings from Sudden Death Victims

Both myocarditis and cardiomyopathies constitute relevant causes of sudden cardiac death in the young population [9,42]. Recent data suggest a relative contribution of 12% for myocarditis, 10% for ACM, and 4% for DCM [43]. Irrespective of the final diagnosis, the autoptic series revealed significant overlapping findings, including inflammatory infiltrates that constantly met definite criteria for myocarditis [6]. 

In DCM, common findings beyond LV volume dilation and wall thinning include myocyte atrophy, vacuolar degeneration, nuclear pleomorphism, and fibrosis [44]. In this setting, it has been suggested that minimal inflammatory foci may be an early sign of inherited cardiomyopathy [23,24]. In many DCM, however, inflammatory infiltrates are composed of lymphocytes, with no evidence of an activated phenotype or myocytolysis [44]. Conversely, positive staining for CD3 labeling the activated phenotype of T-lymphocytes has been mainly reported in classic myocarditis and inflammatory DCM [6]. Similar findings have been described in myocarditis patients known for systemic autoimmune diseases, such as systemic sclerosis [45].

As for ACM, the main histopathological feature is the progressive loss of ventricular myocardium and fibro-fatty replacement [44]. In the LV-dominant forms of ACM, fibrofatty or fibrous scars are typically located in the epicardial layers of the posterolateral free wall [46], mimicking the distribution pattern of classic myocarditis [4,5]. In ACM, myocardial atrophy results from injury and repair processes, which are often accompanied by patchy myocarditis containing CD45^+^ and CD43^+^ T-lymphocytes in up to 75% of hearts at autopsy [47]. Lymphocytic inflammatory infiltrates and focal fibrosis have been reported as concealed substrates even in patients carrying nondesmosomal variants of ACM overlapping with channelopathies such as Brugada syndrome [25,48]. Since myocyte necrosis fulfilling the Dallas criteria [8] is seldom evident on autopsy, the differential diagnosis between true myocarditis and cardiomyopathy-associated M-Infl remains unsolved for many patients [3].

Given the considerable overlap among ACM, DCM, and chronic myocarditis, differential diagnosis should rely on experienced cardiovascular pathologists and integrated clinical-pathological assessment [49].

### 2.3. Overlapping Phenotypes in Sporadic Disease

The clinical presentation of myocarditis is extremely variable and ranges from acute coronary syndrome-like chest pain to heart failure and arrhythmias [6]. In turn, the latter manifestations are frequently found in primary cardiomyopathies: while heart failure is more common in DCM, VA have been described in both ACM [13] and early-stage DCM [12,50,51]. To be noted, however, hot-phase bursts of M-Infl resembling an infarct-like clinical presentation of acute myocarditis have been reported even in ACM patients irrespectively of family history [19,21,22]. Given the complex and widely overlapping scenarios, differential diagnosis is challenging in sporadic diseases.

**Figure 3:**
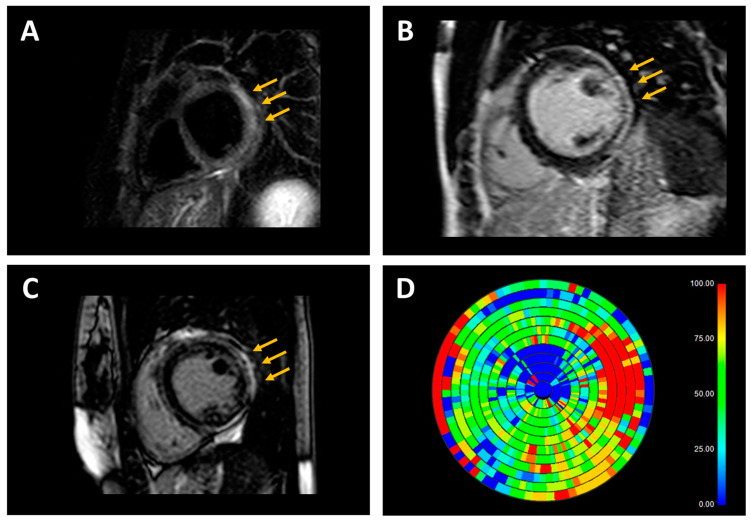
Representative examples of cardiac magnetic resonance (CMR) findings in patients with inflammatory and genetic cardiomyopathies. (**A**) T2-weighted short-tau inversion recovery (STIR) sequence in a patient with acute myocarditis and nonischemic distribution pattern of substrate abnormalities (subepicardial hyperintensity in inferolateral left ventricular wall, arrows). (**B**) Late gadolinium enhancement (LGE) sequence in a patient with genetic dilated cardiomyopathy (DCM; pathogenic variant in the *FLNC* gene) and nonischemic distribution pattern of substrate abnormalities (subepicardial hyperintensity in inferolateral left ventricular wall, arrows). (**C**) LGE sequence in a patient with genetic arrhythmogenic cardiomyopathy (ACM; pathogenic variant in the *DSP* gene) and nonischemic distribution pattern of substrate abnormalities (subepicardial hyperintensity mainly involving the inferolateral left ventricular wall, arrows). (**D**) In the patient with genetic ACM, the LGE map shows an extensive left ventricular scar burden (red = maximal scar burden; blue = healthy myocardium).

In this setting, CMR has been suggested as a valuable diagnostic tool. As a common trait, classic myocarditis, ACM, and DCM present with a nonischemic distribution of substrate abnormalities (Figure 3). However, the presence of LV LGE exceeding the degree of systolic dysfunction, especially when arrhythmic manifestations dominate heart failure symptoms, suggests primary ACM [51]. Furthermore, a ring-like pattern of LGE could better identify primary cardiomyopathies, in particular those secondary to *DSP* and *FLNC* pathogenic variants [52]. On the other hand, septal involvement and associated conduction system defects may point to *LMNA* cardiomyopathy and an adverse prognosis [50,53,54]. 

Once the suspicion of cardiomyopathy is confirmed by CMR, additional effort is needed to identify M-Infl. Very recent data suggest that EMB-proven myocardial inflammation is found in more than 50% of patients with clinically suspected ACM [5]. Consistently, the presence of associated abnormalities at T2-weighted sequences on CMR suggests M-Infl [16]. Additional diagnostic tools allowing M-Infl identification include FDG-PET [55] and ECG recording during arrhythmias. In detail, polymorphic and irregular VA appearances on the 12-lead ECG may point to M-Infl, in contrast with the scar-related regular and monomorphic arrhythmias observed both in healed myocarditis and primary cardiomyopathies [56,57,58]. 

**Figure 4:**
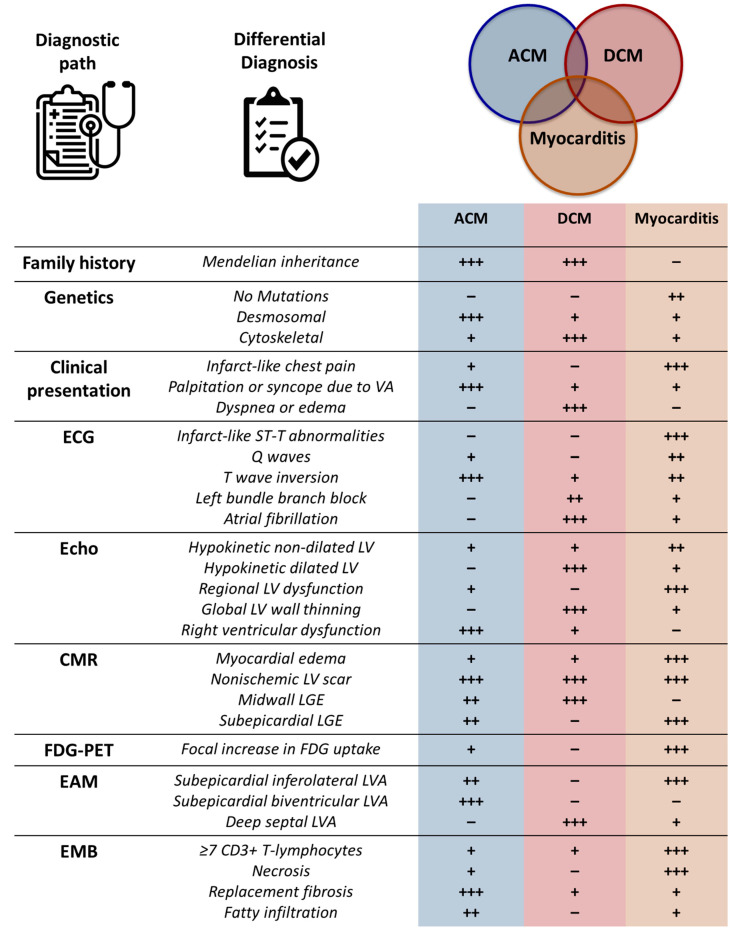
Diagnostic path and differential diagnosis between myocarditis, arrhythmogenic, and dilated cardiomyopathies. Key elements in differential diagnosis between myocarditis, arrhythmogenic, and dilated are shown from a clinical to an instrumental viewpoint. A semiquantitative scale is used to highlight the main differences. ACM = arrhythmogenic cardiomyopathy; CD = cluster of differentiation; CMR = cardiac magnetic resonance; DCM = dilated cardiomyopathy; EAM = electroanatomic mapping; EMB = endomyocardial biopsy; FDG-PET = ^18^F-fluorodeoxy positron emission tomography; LV = left ventricular; LVA = low-volage areas; LGE = late gadolinium enhancement; VA = ventricular arrhythmias.

A complete overview allowing differentiation among myocarditis, DCM, and ACM is presented in Figure 4. As for etiology identification in sporadic disease, genetic testing might be considered in the presence of recurrent myocarditis bursts, persistent LV dysfunction or arrhythmias, or a special LGE pattern on CMR [21,22,52].

## 3. Modeling Myocardial Inflammation in Cardiomyopathies: Evidence from the Preclinical Setting

### 3.1. Molecular Mechanisms of Primary Cardiomyopathies

**Figure 5:**
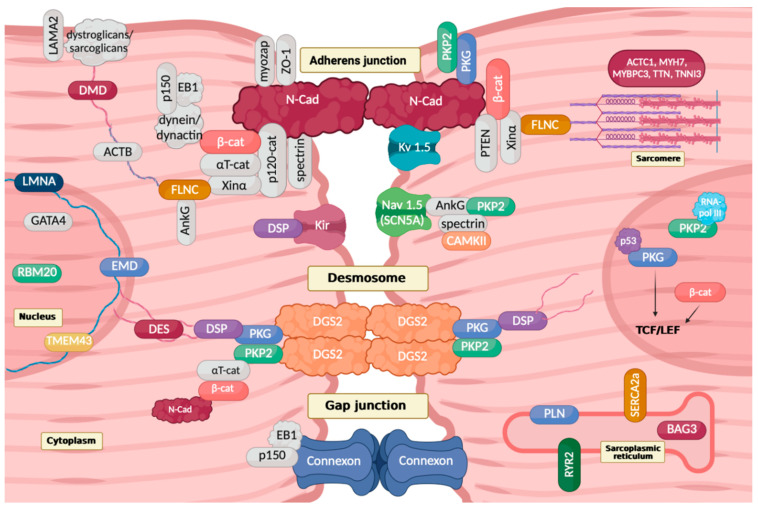
Genetic architecture of genetic cardiomyopathies. The main molecular players involved in the pathophysiology of either arrhythmogenic, or dilated cardiomyopathies are shown, together with the localization of single molecules in cellular compartments.

The main molecular players involved in cardiomyopathy pathophysiology are shown in Figure 5. Given their primary role in determining the architecture and contractile function of cardiac myocytes, genetic variants in a wide variety of proteins of the cytoskeleton, sarcomeres, and calcium handling system have been associated with DCM [59,60]. In detail, DCM phenotypes from structural changes and impaired mechanotransduction are observed secondary to pathogenic variants in a number of cytoskeletal genes, including TTN (titin), *FLNC* (filamin C), *DES* (desmin), *DMD* (dystrophin), *LDB3* (ZASP protein), *BAG3* (BCL2-associated athanogene 3), and the same desmosomal genes involved in ACM pathophysiology [61,62,63,64,65,66]. In addition, variants in genes encoding sarcomeric proteins, such as myosin binding proteins and troponin, or calcium-handling proteins such as phospholamban, encoded by the *PLN* gene, have been associated with DCM phenotypes [67,68].

On the other hand, the electrical uncoupling between cardiac myocytes secondary to pathogenic gene variants involving the intercalated disk components has been proposed as the main arrhythmogenic mechanism in primary ACM [69,70]. With an estimated prevalence of 19–46%, *PKP2* accounts for the majority of variants in adult-onset ACM [71]. As opposed, pediatric cohorts show prevalence of *DSP* [72], where C-terminal variants account for up to 16% of ACM, frequently with a LV-dominant variant [73]. Irrespective of the specific variant, the common pathophysiologic factors observed in ACM [69,74] include plakoglobin redistribution from the membrane to the intercellular pool [75], gap junction remodeling with loss of connexin 43 (Cx43) [75], and apoptosis [76]. Among nondemsosomal proteins, a relevant role is played by ion channels, which reside in the intercalated disks [69]. In particular, pathogenic variants in *SCN5A*, which encodes the pore-forming subunit of the sodium channel Nav1.5, have been associated with a number of arrhythmogenic disorders ranging from isolated channelopathies to structural forms of ACM or DCM [48,77].

Finally, the linker of nucleoskeleton and cytoskeleton (LINC) complex tethers the nuclear envelope to the cytoskeleton and controls the transcription of a broad range of genes, including those implicated in DCM and ACM phenotypes [78]. For instance, the nuclear proteins lamin A and C, encoded by alternative splicing by the *LMNA* gene, account for a life-threatening ACM/DCM with potential multisystemic involvement and age-related penetrance [53,79]. Among the other LINC proteins, while pathogenic variants in *TMPO* have been associated with DCM with a low prevalence of approximately 1% [80], variants in transmembrane protein 43 (*TMEM43*) account for a non-neglectable proportion of ACM cases [81].

### 3.2. Role of Systemic and Local Inflammation

Multiple clues suggest a critical role for both systemic inflammation and M-Infl in cardiomyopathy pathophysiology. For both DCM and ACM, preclinical evidence suggesting a role for inflammation is summarized in Table 3.

In DCM, the cytokine network has been implicated in the pathophysiology of LV dilation and systolic dysfunction. It has been shown that systemic inflammation affects myocardial stiffness by multiple mechanisms, such as post-translational modifications of titin [109]. Remarkably, truncating variants in the *TTN* gene, encoding titin, account for 25% of familial DCM and 18% of idiopathic DCM [110]. Phosphorylation of the N2B segment by protein kinases decreases the distensibility of titin, while phosphorylation of the PEVK-domain has the opposite effect [111]. An arrhythmic phenotype of *TTN*-associated DCM has been described due to protease vulnerability [112]. Consistently, EMB-proven M-Infl has been documented in a patient presenting with severe heart failure and VA [113]. In this setting, titin might play a major role in immunity regulation. In a recent article, DCM patients with and without a truncating *TTN* variant showed an autoimmune/inflammatory trigger in 9% and 12%, respectively [114].

However, gene-environment interactions may be more complex. For instance, it has been shown that toll-like receptor 3 variants increase susceptibility to enteroviral myocarditis and DCM [115]. In patients with immune checkpoint inhibitor (ICI)-related myocarditis and myositis, anti-titin and anti-potassium channel Kv1.4 antibodies are often detected [116]. Remarkably, pathogenic titin variants in solid tumors are associated with higher immunogenicity and inflammatory infiltrates [117]. Recently, it has also been suggested that mimic peptides from commensal bacteria can promote inflammatory cardiomyopathy in genetically susceptible individuals [118]. Consistently, the use of immunosuppressive therapy [119,120], as well as IL-1 inhibitors [121], to target EMB-proven M-Infl, has been associated with LV reverse remodeling and improved systolic function in clinical practice.

As for ACM, high circulating levels of proinflammatory cytokines, as well as reduced levels of anti-inflammatory ones [122], have been reported irrespectively of the specific underlying genetic background. For instance, plakoglobin translocation from intercalated disks to the intracellular pool has been observed in response to low concentrations of proinflammatory cytokines, including IL-6 and TNF-α in granulomatous myocarditis and ACM but not in classic lymphocytic myocarditis [123]. Regardless of the underlying pathogenic variant, one of the main drivers of myocardial inflammation in the ACM is signaling by glycogen synthase kinase-3β (GSK3β) an ubiquitin protein involved in the proteasomal degradation system. In ACM hearts, GSK3β translocates from the cytoplasm to the intercalated disk, resulting in activation of nuclear factor-κB (NFκB), the master cellular regulator of inflammation and innate immune response [95]. Downstream NFκB, transforming growth factor-β3 (TGFβ3) induces a fibrotic response by promoting the expression of extracellular matrix genes and by suppressing the activity of matrix metalloproteinases [124]. As for humoral autoimmunity, anti-DSG2 antibodies have been shown to cause gap junction dysfunction regardless of the ACM genotype [41]. The proarrhythmogenic role of DSG2 autoantibodies is further enhanced by the positive correlation found between circulating levels and the burden of premature ventricular contractions [41]. 

**Figure 6:**
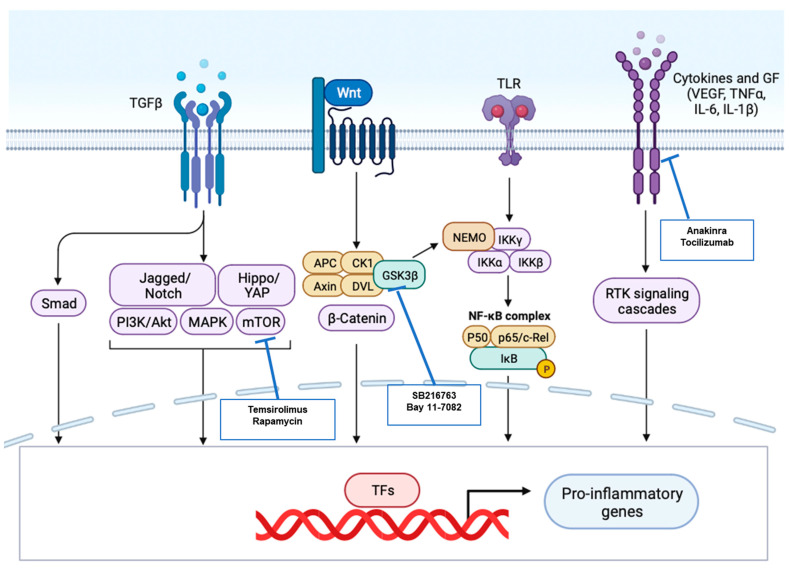
Proinflammatory pathways upregulated in cardiomyopathies and targets for molecular therapy. Suitable molecular targets to mitigate myocardial inflammation in primary cardiomyopathies are shown, along with the proinflammatory pathways most commonly involved. Biological agents and immunomodulatory drugs currently available are in evidence.

Since the whole process of M-Infl is too complex to be properly reproduced in vitro, cell and animal models have been developed to understand the molecular mechanisms of inflammatory cardiomyopathies [125]. In this setting, key targets for biological and immunomodulating therapy are summarized in Figure 6.

### 3.3. Inflammation Models in Genetic Etiologies Linked to DCM

A common pathway for most of the described genetic forms of myocarditis susceptibility is NF-kB. It has been shown that NFκB activation in cardiomyocytes is per se sufficient to cause DCM. Inducible transgenic mice with cardiomyocyte-specific constitutively active IκB kinase (IKK; activator of NF-kB) induce an excessive inflammatory response and myocyte atrophy, leading to a reversible form of heart failure [92].

While *TTN* transgenic animal models have not been investigated for cardiac inflammatory patterns, so far, *TTN* protein has been indirectly linked to viral myocarditis pathogenesis. Indeed, in a model of Coxsackie virus B3 (CVB3)-induced myocarditis, impaired titin phosphorylation was observed, along with LV dysfunction, increased cardiac inflammation, including IL-6 levels, and fibrosis. In this setting, IL-6 receptor blockade led to a reduction in viral load paralleled by extracellular matrix regulation, and titin function improved, resulting in preserved LV function [82]. This concept can be further generalized. Viruses or other infecting agents release proteases that interfere with cytoskeleton/sarcomere proteins, as, for instance, desmin or cardiac troponin, worsening an already compromised situation when these proteins are defective due to a genetic variant [32].

Defects in phospholamban and other proteins involved in Ca^2+^-dependent signaling, have both a direct role in arrhythmia induction as well as in the maladaptive remodeling of the heart [126]. The impaired cellular Ca^2+^ content leads to the abnormal activation of key mediators, such as calmodulin-dependent kinase II (CaMKII) and calcineurin. Active CaMKII, among many functions, is known to increase inflammatory signaling [127]. However, myocardial inflammation has not been investigated in *PLN* animal models.

The RBM20 protein is an essential component of the RNA processing machinery and is required to regulate cardiac gene expression and processing. Using a human-induced pluripotent stem cell (hiPSC)-derived platform, the genes dysregulated by *RBM20* pathogenic variants have been revealed. Among them, 16 are involved in heart diseases, such as cardiomyopathies and inflammation of the myocardium [83].

Dystrophin-deficient rats (Dmd^mdx^) are used to recapitulate the pathological phenotype of DMD patients. The first immunophenotype profile assessed in skeletal and cardiac muscles excised from Dmd^mdx^ rats showed leukocyte infiltration at the age of 12 weeks, which consisted mostly of macrophages and T cells, including CD68^+^ and CD45RC^high^ T cells [84]. Accordingly, another study pointing at the characterization of the cardiovascular phenotype of the Dmd^mdx^ rat demonstrated CD68^+^ macrophage infiltration in both left and right ventricles [85]. Collectively, these data suggest that the recruitment of inflammatory cells could be at the basis of the cardiac dysfunction of DMD patients, who indeed develop a progressive DCM characterized by inflammatory cell infiltration, necrosis, and cardiac fibrosis.

In MyH-mutant mouse hearts, which show heart failure and arrhythmias, the inflammasome pathway was upregulated [86].

It has been shown that expression of mutant *LMNA* (*LMNA*-p. Leu140_Ala146dup) in HEK293 cells led to increased expression of the pro-inflammatory protein Hsp70, mimicking its increase in serum exosomes of patients harboring this same variant [87]. Interestingly, the degree of inflammation, in terms of the amount of pro-inflammatory cytokines upregulated, correlated with the severity of the clinical manifestations associated with each patient. Besides heterologous systems, several mouse models carrying different pathogenic variants of *LMNA* have been obtained, which recapitulate the severe human cardiac structural and arrhythmic defects [128]. One in particular, a transgenic with cardiac-specific prelamin-A accumulation (csPLA-Tg) exhibited a phenotype consistent with inflammatory cardiomyopathy [88]. In the *LMNA* context, it was demonstrated that the trigger for inflammation is likely linked to premature myocardial senescence [129], due to the nuclear lamina disruption, and mediated by the senescence-associated secretory phenotype (SASP) [130]. Inflammaging was sustained by the high myocardial activation of NFκB in csPLA-Tg. Finally, in a *Lmna^CMKO^* mouse model, cardiomyocyte nuclear rupture occurred 2 weeks prior to the development of fibrosis and reduction in ejection fraction and was accompanied by upregulation of pro-inflammatory gene expression and cytoplasmic release of HMGB1, a potent pro-inflammatory protein normally localized in the nucleus [89]. This nuclear-driven pro-inflammatory machinery comes into play in the early stages of the disease, possibly accounting for the development of DCM. Consistently, in animal models, the inhibition of the mTOR pathway by temsirolimus or rapamycin was able to rescue the DCM phenotype [131].

Although cardiac muscle inflammation is minimal in *BAG3*-deficient mice [90], a lack of BAG3 is associated with a pathogen-dependent reaction. Indeed, NF-kB regulates *BAG3* expression in the presence of lipopolysaccharide, the main component of the bacterial membrane, hinting at BAG3s’ implication in the bacteria-induced response, which is associated with enhanced expression of pro-inflammatory cytokines [132]. In addition, a mouse model overexpressing the *BAG3* inhibitor Tat, is more sensitive to viral infection, developing virus-associated cardiomyopathy [133].

The association between myocardial inflammation and cardiac dysfunction in DCM caused by the rare *MYBPC3* variant has been investigated in the cMyBP-C^(t/t)^ mouse model of DCM at 3 months of age [91]. This study described the infiltration of activated (CD45^+^CD11b^+^Ly6C−MHCII^+^F480^+^) and pro-inflammatory M1 (CD45^+^CD11b^+^Ly6C^−^MHCII^+^F480^+^CD206^−^) macrophages, along with upregulation of inflammatory pathways, in the hearts of DCM animals.

### 3.4. Inflammation Models in Genetic Etiologies Linked to ACM

A transcriptome analysis in mice revealed that functional pathways related to viral infection, platelet activation, inflammation, and immune response networks were inversely correlated with *PKP2* expression [94]. *PKP2* haploinsufficiency in the murine heart does not lead to overt phenotypes but has been demonstrated to make the heart more sensitive to experimental autoimmune myocarditis (EAM). The induction of EAM resulted in more subepicardial fibrosis, loss of Cx43 expression, and larger non-myocyte areas [93]. The mouse model with cardiomyocyte-specific conditional homozygous knock-out of PKP2 (*PKP2cKO*), instead, phenotypically shows biventricular dilation and few spontaneous premature ventricular contractions, worsened by isoproterenol induction [134]. Transcriptome analyses revealed, among others, pathways enriched for inflammatory and pro-fibrotic genes. In particular, untranslated region variants in the TGFB3 gene in ACM can lead to increased myocardial fibrosis [135] via activation of the SMAD2/3 and mitogen-activated protein kinase signaling pathways [122,136]. In a mouse derived by crossing *PKP2cKO* with a RiboTag line, *PKP2* loss in cardiomyocytes was transcriptionally linked to genes coding for host-response mechanisms even in the absence of an exogenous trigger [94]. In this model, infiltration of CD45^+^ cells in the sub-epicardial layer of *PKP2cKO*/*RiboTag* mouse hearts was seen in the early phases of the disease [94], hinting at a deficient cardiomyocyte-dependent recruitment of inflammatory cells from the bloodstream. Accordingly, hiPSC-derived cardiac myocytes from a carrier of a *PKP2* variant are characterized by an over-activation of NFκB and increased cytokine expression [95], describing a cardiac activation of innate immunity. In this setting, small molecules such as the GSK3β inhibitor SB216763 and Bay 11-7082 have shown the capability of reversing the ACM phenotype [94,95,99,100]. In particular, SB216763 normalized the expression and localization of GSK3β as well as plakoglobin and Cx43 with positive effects on arrhythmogenesis [122].

In mice with cardiac-restricted inactivation of wild-type desmosomal cadherins, as well as in those with overexpression of mutant ones, inflammatory macrophagic infiltration has been described in nearby necrotic tissue [70,96,137]. In the first-generated mouse model of *Dsg2*-related right ventricular ACM (a transgenic mouse overexpressing dsg2-N271S), the progression of the disease phenotype has been finely reported [96]. It involves: (i) necrotic cell death that prompts an inflammatory response, mediated by massive inflammatory infiltrates, and calcification in the myocardium, (ii) injury repair with fibrous tissue replacement. Early activation of inflammatory-associated pathways, along with upregulation of genes linked to specific macrophage populations, was confirmed also in other *Dsg2* mouse models [97], further strengthening the idea that activation of the pro-inflammatory machinery plays a pivotal role in generating the phenotype in *Dsg2* mouse models of ACM. In addition to this, the contribution of inflammatory cells to all stages of murine ACM has been reported and comprises neutrophil granulocyte recruitment at the disease onset, recruitment and differentiation of macrophages during acute disease progression, and persistence of T cells during chronic disease progression [98]. The increased levels of inflammatory cytokines and chemotactic molecules were associated with activation of NFκB signaling, in a mouse model in which exons four and five of Dsg2 were excised to generate a frameshift variant [95]. Consistently, treatment of *DSG2* mutant mice with Bay 11-7082 reduced the number of infiltrating inflammatory cells in the myocardium [95].

While *DSC2* KO mice do not show a cardiac phenotype unless stressed [138], cardiac-specific overexpression of wild-type DSC2 causes biventricular cardiomyopathy. This includes severe fibrosis, cardiac necrosis, calcification, and cardiac inflammation [104]. Accordingly, gene expression data indicate up-regulation of genes responsible for acute sterile inflammation (cytokines, chemokines, and toll-like receptor signaling) and fibrotic remodeling [104].

Neonatal rat ventricular myocytes transfected to express the variant *2057del2* of plakoglobin showed typical cardiomyocyte-specific ACM phenotypes, including the release of different inflammatory mediators into the culture medium, such as IL-6, TNF-α, and MIP-1α [100]. These features were normalized when cells were incubated with the GSK3β inhibitor SB216763 [100,101]. In addition, the transfected cardiomyocytes with the *JUP2157del2* construct showed nuclear signal for phospho-RelA/p65 (Ser536), indicating activation of NFκB, which was prevented by the anti-inflammatory Bay 11-7082. Accordingly, inflammatory cells, mostly neutrophils and macrophages, were observed in homozygous *Jup* mutant mice obtained by ablating Jup in cardiomyocytes. This agrees with the increased expression of pro-inflammatory cytokines IL-1β and IL-6 in Jup mutant hearts [102]. Among WNT pathway mediators, stromal progenitor cells from the hearts of plakoglobin transgenic mice (truncation variant of *Jup*) overexpressed IGFBP5, which has many biological functions, including in the inflammatory response [103,139].

A cardiac-specific *Dsp* knockout in the heterozygous form was obtained in mice. Haploinsufficiency gave rise to fibro-fatty substitution of the myocardium and cardiomyocyte death, leading to cardiac dysfunction and arrhythmogenesis [140]. Interestingly, among the differentially expressed genes for WNT pathway inhibitors and endothelial to mesenchymal transition mediators, those encoding for inflammatory proteins were highly expressed in the cardiomyocytes of these mice [140].

The hearts of *Tmem43-S358L* mutant mice are characterized by NFκB activation. However, this activation does not promote a typical cardiac inflammatory response, instead leading to TGFβ-mediated fibrosis [105]. On the other side, inflammatory cell infiltration was also observed in homozygous *Tmem43-S358L* KI rats [106]. Consistently, GSK3β inhibition in *TMEM43* mutant mice resulted in a relative improvement in the contractile function [105,141].

Other animal models with different but still unknown genetic causes phenotypically display a cardiomyopathy with an inflammatory aspect. ACM in boxer dogs is inherited, but the genetic etiology is still debated [142,143]. Dogs show ventricular arrhythmia, ventricular dilation, fibro-fatty replacement, and myocarditis, characterized by patchy mononuclear cells infiltrating the RV [107]. Auto-antibodies against DSG2, proposed as a specific biomarker for all genetic forms of ACM and as a proof of autoimmunity resultant from ACM pathogenic gene variants, have been found in the sera of boxer dogs spontaneously manifesting the disease [41], but were never tested in ACM mouse models. Spontaneous feline ACM, characterized by right-ventricular disease, ventricular tachycardia, and fibro-fatty replacement of the myocardium, also has signs of inflammatory infiltrates [108].

## 4. Clinical Implications and Conclusive Remarks

To date, the clinical significance of M-Infl in genetic cardiomyopathies is still under investigation. Preliminary evidence suggests that the detection of M-Infl by multimodal workup is feasible and allows the subsequent use of immunomodulatory treatment [144]. However, the impact of immunomodulatory strategies on outcomes is still controversial. For instance, while M-Infl has been recognized as a major pathophysiological contributor to heart failure, the results of several trials attempting anti-inflammatory strategies in uncharacterized DCM did not result in a significant clinical improvement [145]. As opposed, in patients with EMB-proven inflammatory DCM, the use of immunosuppressive therapy has been proven safe and associated with LV reverse remodeling and recovery of the systolic function [6,119]. In light of its reversibility potential, M-Infl has been proposed as an indicator of good prognosis even in pediatric series [146]. In the setting of DCM, the use of IST is meant not only to prevent end-stage heart failure but also to avoid the improper application of implantable cardioverter defibrillators in primary prevention [4,147]. As for the arrhythmic manifestations, it has been suggested that M-Infl is capable of predicting ventricular arrhythmia recurrences in patients with classic lymphocytic myocarditis [58], as well as in cases with M-Infl in the context of rare genetic variants of the DCM/ACM spectrum [5]. In both conditions, preliminary data support a favorable role of immunosuppressive therapy on arrhythmic outcomes [5,39,144]. In light of the above, a careful assessment of the myocardial inflammatory status may result in patient-tailored strategies to prevent sudden arrhythmic death [4,148]. Additional evidence from larger multicenter trials is needed before such preliminary evidence can be translated into practical clinical guidelines.

In conclusion, based on current knowledge, M-Infl is expected to play a major role in the pathophysiology of cardiomyopathies. In detail, both clinical and preclinical evidence support multiple interconnections between cardiac inflammation and diseases of the DCM and ACM spectrum. As shown in Figure 4, genetic testing should be more extensively applied to distinguish specific etiologies among a spectrum of cardiomyopathies with overlapping phenotypes [149]. On the other hand, further studies using in vitro and in vivo models are needed to explore the causal relationship between M-Infl and inherited cardiomyopathy genes [150]. In this setting, inflammation could also be a general, aberrant, and unspecific response to the impairment of cardiomyocyte function, irrespective of the underlying etiology, microbial, chemical, or primarily genetic. The contribution of human leukocyte antigen (HLA) variants and immune genes deserves appropriate investigation as well. Finally, additional evidence is expected from the pharmacological targeting of inflammation in genetic diseases. Preliminary data from animal models [95,122] and clinical experience [5,39,119] showed that the use of anti-inflammatory and immunomodulating therapy was accompanied by an improvement in both the arrhythmic and mechanical manifestations of the diseases. These findings pave the way for novel therapeutic perspectives and trials in human disease.

## Figures and Tables

**Table 1:** Inflammatory cardiomyopathies: key concepts and definitions.

Term	Definition	References
Myocarditis	Inflammatory disease of the myocardium diagnosed by established Histological, immunological, and immunohistochemical criteria (WHO, ESC). In detail: -Histology: infiltrating inflammatory mononucleated cells with myocyte myocyte degeneration and necrosis of nonischemic origin (Dallas criteria). It is defined borderline myocarditis in the absence of necrosis. It is defined chronic myocarditis in the presence of replacement-type fibrosis. -Immunohistochemistry: ≥14 leucocytes/mm^2^ including up to 4 monocytes/mm^2^ with the presence of CD 3 positive T-lymphocytes ≥7 cells/mm^2^. Clinical classification based on symptom onset: -Acute (<1 month) -Subacute (1–3 months) -Chronic (>3 months)	[6,7,8]
Cardiomyopathy	Myocardial disorders in which the heart muscle is structurally and functionally abnormal, in the absence of coronary artery disease, hypertension, valvular disease and congenital heart disease sufficient to cause the observed myocardial abnormality (ESC). It may also include electrical diseases prone to life-threatening arrhythmias (AHA).	[9,10,11]
Dilated cardiomyopathy (DCM)	Dilation and impaired contraction of the left or both ventricles that is not explained by abnormal loading conditions or coronary artery disease. Phenotype classification:-Hypokinetic nondilated cardiomyopathy (LV systolic dysfunction with no dilation). -Overt DCM (LV dilation and systolic dysfunction).	[6,12]
Arrhythmogenic cardiomyopathy (ACM)	Arrhythmogenic heart muscle disorder not explained by ischemic, hypertensive, or valvular heart disease. Phenotype classification: -Classic right ventricular ACM (modified Task Force Criteria). -Biventricular ACM. -Left-dominant ACM (Padua criteria).	[13,14]
Inflammatory cardiomyopathy	Myocarditis in association with cardiac dysfunction (i.e., LV ejection fraction <50%)	[6,7]
Myocardial inflammation (M-Infl)	Evidence of myocardial inflammation fulfilling the histological (±myocyte necrosis, i.e., borderline myocarditis) and immunohistochemical criteria for myocarditis, in a patient with clinical diagnosis of ACM or DCM.	[3,5]

Common definitions in the field of myocarditis and cardiomyopathies are shown. ACM = arrhythmogenic cardiomyopathy; AHA = American Heart Association; CD = cluster of differentiation; DCM = dilated cardiomyopathy; ESC = European Society of Cardiology; LV = left ventricular; M-Infl = myocardial inflammation; WHO = World Health Organization.

**Table 2:** Evidence of myocardial inflammation in human cardiomyopathies.

Gene	Model	Main Findings	References
*DSP*, *PKP2*	Clinical setting	Patients with *DSP* variant cardiomyopathy including 16/105 (15%) who had “acute myocardial injury episodes” akin to clinical myocarditis.	[18]
*DSP*	Clinical setting	Acute myocarditis reflects an active phase of ACM that leads to changes in phenotype and abrupt progression of ACM.	[19]
*DSP*	Clinical setting	Cohort of patients initially presenting with a classic myocarditis syndrome (chest pain, troponin elevation) who were subsequently diagnosed with ACM. Most patients had a *DSP* genetic variant.	[20,21,22]
*DSP*, *LAMA4*, *LDB3*, *MYBPC3 DSC2*, *RYR2*, *SOS1*, *SCN5A*, *SGCD*, *LPL*, *PKP2*, *MYH1*, *GATA6*, *and DSG2*	Human heart specimens from autopsy cases	Minimal inflammatory foci may be an early sign of inherited cardiomyopathy.	[23]
*DSP*, *FLNC*, *PKP2*, *TMPO*, *TTN*	Human EMB	Retrospective multicenter study on patients with undefined LV ACM and extensive overlap between EMB-proven myocardial inflammation and rare genetic variants of the DCM/ACM spectrum.	[5]
	Human EMB	Most asymptomatic relatives of dilated cardiomyopathy patients with mild left ventricular enlargement already showed infiltration of inflammatory cells, at levels that were similar to those of patients with established disease.	[24]
*SCN5A*	Case report	Young SCN5A variant carrier with recurrent ventricular fibrillation and massive myocardial inflammation	[25]

The main reports linking myocardial inflammation to primary cardiomyopathies in human subjects are shown.

**Table 3:** Preclinical models of inflammatory cardiomyopathies.

Models of Genetic Etiologies Linked to DCM			
Gene	Model	Phenotype	References
*TTN*	C57BL6/J mice	Cardiac inflammation	[82]
*RBM20*	shRbm20 iPSCs	Dysregulation of genes involved in inflammation	[83]
*DMD*	Dmd^mdx^ rat	Infiltration of leukocytes	[84]
*DMD*	Dmd^mdx^ rat	Infiltration of macrophages	[85]
*MYH*	MyH-mutant mouse	Upregulation of inflammasome pathways	[86]
*LMNA*	HEK293 cells expressing LMNA-p. Leu140_Ala146dup	Upregulation of Hsp70	[87]
*LMNA*	csPLA transgenic mouse	Inflammatory cardiomyopathy; activation of NF-κB	[88]
*LMNA*	*Lmna^CMKO^* mouse	Upregulation of pro-inflammatory gene expression programs	[89]
*BAG3*	BAG3-deficient mice	Minimal cardiac muscle inflammation	[90]
*MYBPC3*	cMyBP-C^(t/t)^ mouse	Macrophage infiltration; upregulation of inflammatory pathways	[91]
*-*	IKK^MyHC^ mouse	Excessive inflammatory response and myocyte atrophy	[92]
**Models of Genetic Etiologies Linked to ACM**			
**Gene**	**Model**	**Phenotype**	**References**
*PKP2*	PKP2-Hz mouse	More sensitivity to experimental autoimmune myocarditis	[93]
*PKP2*	C57BL/6 RiboTagflox mice	Abundance of transcripts involved in the inflammatory/immune response	[94]
*PKP2*	hiPSC line from an ACM patient with a c.2013delC (p.Lys672Argfs*12) variant in plakophilin-2 (PKP2)	Secretion of inflammatory cytokines	[95]
*DSG2*	N271Sdsg2 transgenic mouse	Presence of massive inflammatory infiltrates	[96]
*DSG2*	Dsg2^+/+^ mouse, Dsg2^+/−^ mouse, Dsg2^−/−^ mouse	Activation of inflammatory pathways; upregulation of genes linked to specific macrophage populations	[97]
*DSG2*	Dsg2^MT^ mouse, Dsg2^cKO^ mouse	Presence of inflammatory response, recruitment of immune cell	[98]
*DSG2*	Dsg2^mut/mut^ mouse	Activation of NFκB; increased levels of inflammatory cytokines and chemotactic molecules	[95]
*DSC2*	DSC2 transgenic mouse	Upregulation of inflammatory and fibrotic remodeling pathways	[99]
*JUP*	Neonatal rat ventricular myocytes expressing 2057del2 plakoglobin	Release of inflammatory mediators, reduced by SB216763 treatment	[100]
*JUP*	Dsg2^mut/mut^ mouse, JUP^2157del2^ mouse	Extensive inflammation, improved by SB216763 treatment	[101]
*JUP*	Neonatal rat ventricular myocytes expressing *JUP^2157del2^*	Activation of NFκB	[95]
*JUP*	JUP mutant mouse	Expression of pro-inflammatory cytokines	[102]
*JUP*	PG^TR^ mouse	Overexpression of IGFBP5	[103]
*DSP*	Cardiac-specific *Dsp* knockout mouse	Fibro-fatty substitution of the myocardium; cardiomyocyte death	[104]
*TMEM43*	*Tmem43-S358L* mutant mice	Activation of NFκB	[105]
*TMEM43*	*Tmem43-S358L* KI	Infiltration of inflammatory cells	[106]
*-*	Boxer right ventricular ACM	Myocarditis	[107]
*-*	Cat right ventricular ACM	Signs of inflammatory infiltrates	[108]

Cell and mouse models linking inflammatory mechanisms to ACM and DCM are presented.

## Data Availability

Not applicable.

## Abbreviations

ACM = arrhythmogenic cardiomyopathy; CMR = cardiac magnetic resonance; DCM = dilated cardiomyopathy; EMB = endomyocardial biopsy; FDG-PET = ^18^F-fluorodeoxypositron emission tomography; LGE = late gadolinium enhancement; M-Infl = myocardial inflammation; VA = ventricular arrhythmias.
